# Property Tax Foreclosure, Spatial Effects, and Neighborhood Racial Demographic Change: Examining Data From Detroit

**DOI:** 10.3389/fsoc.2021.598911

**Published:** 2021-06-03

**Authors:** Karl Vachuska

**Affiliations:** Department of Sociology, University of Wisconsin-Madison, Madison, WI, United States

**Keywords:** spatial effects, Property Tax Foreclosure, property abandonment, racial segregation, demographic change

## Abstract

In recent decades, the city of Detroit has experienced the greatest population loss of any major American city. Applying Event History Analysis methodology to a large dataset containing information on all properties in Detroit between 2002 and 2013, I examine how Property Tax Foreclosure spatially perpetuated itself in Detroit, finding evidence that the number of past but recent Property Tax Foreclosures in a localized area significantly predicts the likelihood of a future foreclosure. I extrapolate these findings to mathematical simulations and find evidence that suggests that initial Property Tax Foreclosures played a significant role in cascading many later on. Finally, building off past research that suggests neighborhood blight disproportionally affects white residential preferences and patterns, I perform an empirical analysis that examines how the initial distribution of Property Tax Foreclosures in Detroit neighborhoods played some role in determining how those neighborhoods have experienced racial demographic change.

## Introduction

Detroit, Michigan, serves as an extreme example of a major American city whose property stock has been increasingly subject to vacancy and blight in recent decades. For the last few decades, the city's population has fallen at an increasing rate, and today a sizable percentage of the city's properties sit vacantly. While past research has found that foreclosures of various forms tend to be spatially clustered and that foreclosures may have negative impacts on nearby homes (such as declines in home values), less research has articulated time-wise how Property Tax Foreclosure spatially perpetuates itself and how Property Tax Foreclosure differentially impacts neighborhoods of varying racial compositions (Whitaker and Fitzpatrick, 2013; Gutiérrez and Domènech, 2017).

Property Tax Foreclosure constitutes an instance wherein a property owner has failed to pay the due property taxes on a property for a certain number of years. After this particular number of years, the local government forecloses upon the property and takes ownership of it. In Detroit (from which data in this study comes), Property Tax Foreclosure occurs after three consecutive years of failing to pay property taxes. In this study, I utilize a large dataset containing address and geographical information on all properties in the city of Detroit in addition to data on all Property Tax Foreclosures in Detroit from 2002 and 2013. I apply Cox Proportional Hazard Models to analyze the relationship between local conditions and the likelihood of a property experiencing Property Tax Foreclosure. I find evidence of Property Tax Foreclosure having subtle spatial effects, including an effect of the Property Tax Foreclosure itself, as well as possibly an effect of the presumed earlier vacancy of the property. Utilizing simulations, I extrapolate these findings to a larger scale, which suggests that a critical predictor of the long-term number of Property Tax Foreclosures in a neighborhood is how many initial Property Tax Foreclosures there are throughout the neighborhood. Finally, I perform an empirical analysis and find some evidence that the number of initial Property Tax Foreclosures and how dispersed they were predicted how neighborhood racial demographics shifted in Detroit between 2000 and 2017.

## Background on Detroit

The city of Detroit, Michigan, has undergone significant economic and demographic changes throughout the end of the 20th century and the beginning of the 21st century. Detroit has experienced a significant population decline, particularly for a major city, losing 61% of its population between 1950 and 2010 (Dewar et al., 2015). Several causes are implicated in this significant change, one of which is the increased efficiency of labor in manufacturing, which reduced residents' economic opportunities at an enormous scale, driving residents from the city, further reducing other economic opportunities. Between 2000 and 2017, around the time this paper examines, many large Detroit employers experienced bankruptcy, including the city itself. Additionally, the population of the city fell by ~300,000 during that period.

Economic despair plays a large role in Detroit and creates barriers to sustainable homeownership for many Detroiters. In Michigan, low-income homeowners can qualify for partial or total Property-Tax exemption but need to apply to receive it, and this policy is not very well-known, resulting in <12% of homeowners that qualify receiving it (Eisenberg et al., 2018). As such, if a homeowner in the state of Michigan has property taxes due and fails to pay property taxes for two consecutive years, a county treasurer notifies them that they are at risk of losing their property if the taxes for the third year are not paid by March 31st of the following year. If this requirement is not met, the home is put up for auction, with the minimum bid being the sum of outstanding taxes, interest penalties, and any liens against the property. If the property is not sold at this first auction, a second is held 30 days later, with the opening bid being $500. Notably, Michigan state law makes evictions more complicated. A new homeowner must go to a court at least twice to obtain legal orders permitting them to enter and remove the previous homeowners' person and belongings (Akers and Seymour, 2019).

Numerous contextual factors consequently predisposed paying property tax to be overly burdensome in Detroit. In 2002, budget issues caused property assessment in the city of Detroit to be underfunded, resulting in vast overestimation of property values in Detroit's city. By 2010, it was estimated as many as 85% of homes in Detroit were overvalued. It has further been estimated that around 10% of all Property Tax Foreclosures between 2009 and 2019 resulted from this overvaluation and subsequent overtaxing (Atuahene and Berry, 2019). Notably, wealthier homeowners hired lawyers or real estate experts to appeal the overvaluation and have it reduced, this rarely was attempted by lower-income homeowners. This suggests over-taxation specifically burdened already-poor Detroit homeowners. Besides large numbers of Property Tax Foreclosures, during the period this analysis covers, Detroit was additionally subject to many foreclosures by a mortgage default, as many Detroiters became unemployed due to the economic crisis (Rugh and Massey, 2010).

**Figure 1:**
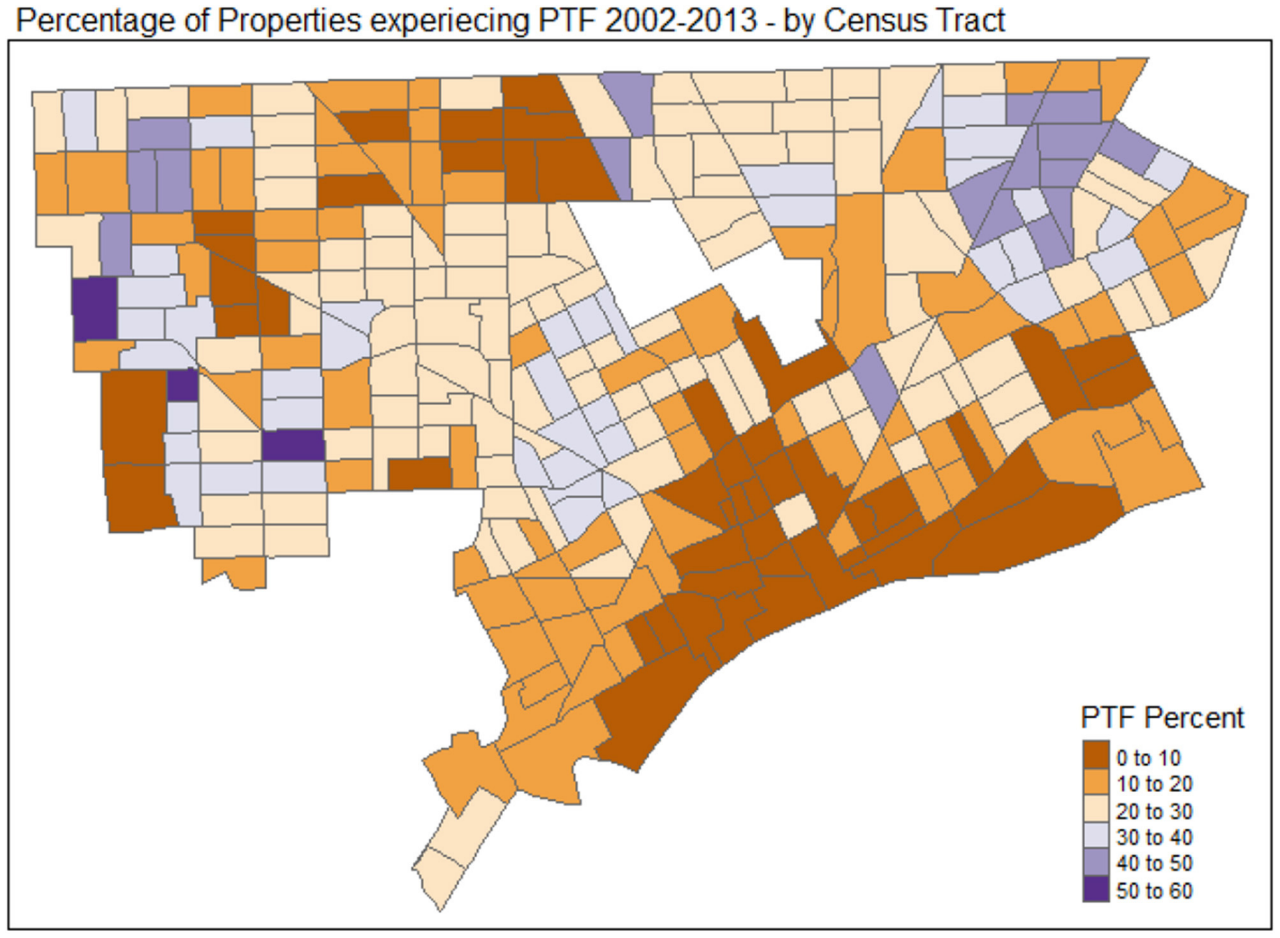
Property tax foreclosure thematic map (Tennekes, 2018).

Ultimately, past research has already highlighted some outcomes of how Property Tax Foreclosures played a significant role in how the city has continued to change since 2000. Dewar et al. (2015) suggest several implications of the Property Tax Foreclosure process in Detroit, which was designed to recover lost Property Tax revenue and reduce the number of vacant properties in the city. In Detroit's case, Dewar et al. describe Property Tax Foreclosure as often indicating that a property owner has walked away from a property, effectively abandoning it. Dewar et al. additionally state that one of the effects of the Property Tax Foreclosure is increasing nearby properties' likelihood of becoming abandoned/experiencing Property Tax Foreclosure. While over one-hundred thousand have been carried out in Detroit since 2000, in 2015, after the period for which data is analyzed in this study, housing policy was changed in Detroit in order to reduce the number of Property Tax Foreclosures in Detroit by offering delinquent homeowners the option to retain ownership as long as they make monthly payments on the overdue Property Taxes. This paper thus specifically examines the effects of Property Tax Foreclosure in Detroit between 2002 and 2013 when they were at their peak, and more strict policy was in place for foreclosing on delinquent property taxpayers. Figure 1 depicts a thematic map portraying Property Tax Foreclosure's frequency in different census tracts in Detroit.

## Literature Review

Massey's (1990) work examined how neighborhoods change under similar conditions of segregation and manufacturing decline. Via simulation on an artificial city, Massey demonstrated that even modest racial and class segregation levels could concentrate poverty in particular neighborhoods. This concentration of poverty has critical effects under the economic transformations and sweeping changes that many large American cities experienced as the manufacturing industry declined and had disproportionate effects on particularly susceptible neighborhoods. Massey performs an empirical analysis finding that neighborhoods with increasing poverty rates deal with many growing associated issues, including growing crime and violence levels, lower standardized test scores, higher dropout rates, and declining housing quality. As Massey's research demonstrates, neighborhoods are particularly susceptible to large-scale economic changes.

While Property Tax Foreclosure can mean multiple things, as Dewar et al. indicated, Property Tax Foreclosure in Detroit's case in many cases, means that property was abandoned and sits vacant. Vacant properties have been documented to have many effects on the area surrounding them. Research has found that vacant property has a localized effect on violent crime, graffiti, the likelihood of fires, and reduces nearby property values and overall neighborhood vitality (Accordino and Johnson, 2000).

Whitaker and Fitzpatrick (2013) examined the impact of Property Tax Foreclosures in Cleveland on nearby properties and their values. Utilizing a radius of 500 feet, they find that a Property Tax Foreclosure has a significant effect on nearby properties' value, reducing sales prices by 1–2%. Distinctly, they find that this effect is much more significant in lower-poverty neighborhoods, where a Property Tax Foreclosure can reduce sales prices by between 4 and 8%. This research suggests Property Tax Foreclosures have a significant effect on the desirability of residing in the immediate vicinity and that this effect is stratified around the neighborhood's socioeconomic status.

Whitaker and Fitzpatrick's research is also critical in examining and discussing the nuance between occupied and foreclosed properties and vacant and foreclosed properties. They find that while both types of Property Tax Foreclosures harm nearby property values, Vacant and Foreclosed properties have a much more significant negative effect than Occupied and Foreclosed properties. The authors attribute this to the idea that Vacant properties are more likely to be poorly maintained and thus “not contribute to the vibrancy or security of a neighborhood.” When an occupied property experiences Property Tax Foreclosure, it may affect the surrounding area in multiple ways since Property Tax Foreclosures are somewhat visible events, meaning the home's pending vacancy may be visible as well as that Property Tax Foreclosure involves appraisal which directly affects the value of neighboring properties.

Beyond Whitaker and Fitzpatrick's (2013) work, which suggests that the economic status of a neighborhood relates to the particular effects of a Property Tax Foreclosure, substantial evidence suggests racial composition, which is often conflated with the socioeconomic status of a neighborhood, has significant effects on neighborhood desirability. Distinctions in neighborhood racial composition preferences, such as whites typically preferring all-white neighborhoods and blacks typically preferring half-white, half-black neighborhoods, are commonly implicated in the presence of racial residential segregation (Krysan and Farley, 2002).

Krysan et al. (2009) utilize a video experiment to examine race and residential neighborhood preferences. They find that neighborhood quality and racial composition have a unique interaction related to whites' neighborhood preferences. While in general, both whites and blacks prefer middle and upper-class neighborhoods with visibly higher-quality housing, whites' preference for residing in lower-working class and blemished middle-class neighborhoods varied substantially based on the racial composition of the neighborhoods. In these lower-working class and blemished middle-class neighborhoods, where some properties are depicted as appearing vacant, whites were much more likely to prefer to live in these neighborhoods if they were all-white as opposed to being mixed (half-black, half-white). Whites were further much more likely to prefer to live in a mixed-neighborhood if it appeared middle or upper class, as represented in the videos by fewer boarded-up homes and less trash on home's lawns. These preferences are further particularly strengthened or reduced based on individuals' unique stereotypes of Black Americans or Black American neighborhoods to begin with.

Stipak and Hensler (1983) implicate the socioeconomic status of Black Americans in residential segregation. While White Americans and Black Americans have different racial neighborhood preferences, White Americans and Black Americans have virtually identical preferences in other qualities. Stipak and Hensler find that a mere four characteristics can explain 65% of total neighborhood preferences: “(1) safety for the respondent, (2) available recreational facilities, (3) quality of the local public schools, and (4) an overall evaluation of the neighborhood as a place to live” Stipak and Hensler argue that Black Americans rarely have these preferences satisfied in their residential decision-making as a result of being unable to afford housing in such neighborhoods. As it relates to this paper, this work suggests Black Americans compared to White Americans, may be less likely to move from blighted neighborhoods and may be more likely to move into them due to having fewer financially-viable residential alternatives.

Past research suggests much about how Property Tax Foreclosures may affect neighborhoods in terms of demographic change. Neighborhoods with more Property Tax Foreclosures should, in general, have lower overall desirability based on the neighborhood qualities that tend to be associated with Property Tax Foreclosures. Given this, and that Black Americans tend to be less successful in satisfying neighborhood preferences based on objective non-racial qualities, I would expect Property Tax Foreclosures to lead to racial demographic change by retaining and adding more black residents relative to the number of retained/new white residents.

In general, the literature suggests some hypotheses for my subsequent analysis. Based on Dewar et al. (2015) statement that Property Tax Foreclosures perpetuates itself as well as Whitaker and Fitzpatrick (2013) localized effect of Property Tax Foreclosure on nearby property values, I can expect the number of nearby properties that have experienced Property Tax Foreclosure to increase the probability that a property experiences Property Tax Foreclosure. Based on Whitaker and Fitzpatrick's finding that lower-poverty neighborhoods are more susceptible to the effects of Property Tax Foreclosures, I can expect that any Property Tax Foreclosure may be more influential in spatially perpetuating Property Tax Foreclosure in whiter neighborhoods since whiter neighborhoods tend to have higher overall socioeconomic status. Building directly off this, since white residents typically have better resources with which to satisfy their neighborhood preferences, I can expect the number of Property Tax Foreclosures in a neighborhood to predict the racial demographic change the neighborhood.

## Data and Methods

### Data

The data for this study comes from two files within the City of Detroit's Open Data Portal. The first file is the City of Detroit's “Motor City Mapping citywide property survey” which contains location data on all unique properties within the City of Detroit in 2013. The second file is “Archival Tax Foreclosures in Detroit, 2002-2013,” which contains data on all Property Tax Foreclosures in Detroit between 2002 and 2013 (Data Driven Detroit, 2019a,b). Merging these two datasets, I am able to create a dataset of all properties in Detroit, with further information on whether or not the property experienced Property Tax Foreclosure between 2002 and 2013, and if so, in which year it did. My dependent variable is the time from 2001 until Property Tax Foreclosure. Duration is measured discretely in years since Property Tax Foreclosure can occur only once a year. Using Event History Analysis methodology, I define when a property experiences a Property Tax Foreclosure as a “failure.” I right censor all properties that do not experience Property Tax Foreclosure.

Since spatial characteristics are of primary interest in this analysis, the composite dataset lends itself to several interesting potential analysis. I have information on the exact geographical position of properties and information on what particular “block” properties are located from addresses. I operationalize “blocks” as a particular range of numbers on a particular street. Every set of hundred numbers is a different block (300–399 is a single range of possible address numbers for a particular block). Utilizing this, I can analyze the effect of the proportion/absolute number of properties on the block that experienced Property Tax Foreclosure and the subsequent probability that a given property will experience Property Tax Foreclosure sooner. My data is in long-form since some of the variables vary with time. Table 1 displays the total number of Property Tax Foreclosures in Detroit by year, and Table 2 display summary statistics on variables used in the analysis.

**Table 1:** Property Tax Foreclosures by year.

**Year**	**Number of Property Tax Foreclosures**
2002	279
2003	2,566
2004	2,067
2005	2,148
2006	5,143
2007	2,347
2008	4,218
2009	8,330
2010	12,104
2011	14,304
2012	22,747
2013	21,003

*Dataset totaled 383,491 unique properties*.

**Table 2:** Summary statistics of covariates.

**Variable**	**Median**	**Mean**	**SD**
Percent (fraction) of Block Properties experiencing PTF in given year	0	0.02113	0.1438307
Total number of properties on block	21	20	10.11433
% Non-Hispanic white in census tract	3.8	10.18191	13.99501
% Black in census tract	93.97	82.51101	24.42768
Percent of vacant housing units for rent in census tract	28.36	30.79156	13.0105
Percent of vacant housing units for sale in census tract	7.33	11.75697	10.74693
Percent of occupied housing units owner-occupied in census tract	56.13	56.92175	16.70999
Percent of occupied housing units renter-occupied in census tract	43.87	43.07825	16.70999
% of housing units with mortgage exceeding 30% of owner's income in census tract	15.66	16.18569	8.148718
% of housing units with mortgage exceeding 50% of owner's income in census tract	7.02	7.872481	5.620022
Renter-specified housing units rent as percentage of income in census tract	23.24	23.30433	7.01557
Percent of population in poverty in census tract	23.97	23.4253	10.55619
Percent of workers employed in manufacturing in census tract	18.76	19.18474	4.938029
Employment rate in census tract	85.52	84.88272	6.316376

### Methods

#### Cox Model

I examine the effects of the measured covariates on Property Tax Foreclosure using a Cox Proportional Hazards Model. The Cox Proportional Hazards Model is a type of statistical survival model that relates the duration of time until an event occurs with a set of covariates, in a matter such that the individual effect of a unit increase in a covariate multiplicatively affects the Hazard rate. A Cox Proportional Hazards Model is appropriate for the dataset and is very practical. The completed periods of time until Property Tax Foreclosure were measured as the number of years between 2001 and Property Tax Foreclosure. Censored periods were 12 years. The Cox model takes the following form:

$$\begin{array}{l} h\left(t\right)=h_{0}\left(t\right)exp\left(X\beta \right) \end{array}$$

where *h* (*t*) is the hazard of Property Tax Foreclosure at yearly duration *t*, *h*_0_(*t*) is an unspecified baseline hazard rate at Property Tax Foreclosure duration t that is affected by the vector of measured covariates **X**.

Detroit experienced several economic shifts between 2002 and 2013 and was affected significantly by the Great Recession. Effects of this include a substantial population decline and notably significant year-to-year variation in the number of Property Tax Foreclosures. I, therefore, have decided to control for this in my analysis by including an indicator variable for year in my model.

For this analysis, it was also important to control external factors that may contribute to properties' likelihood of experiencing Property Tax Foreclosure. Utilizing 2000 Census data, I controlled for a number of neighborhood demographic, economic, and property qualities that may affect Property Tax Foreclosure's likelihood. A list of these control variables is included in Table 2. Specifically, economic factors such as unemployment, employment in the Manufacturing industry, and income relative to mortgage payments and relative to rent predispose foreclosure, so I included these control variables. Additionally, the housing stock's initial qualities, such as the number of vacant housing units and the percentage of housing stock owner and renter-occupied, predispose foreclosure, so I also included some of these variables as controls.

The presence of blighted or abandoned properties is often tied to reduced home values and lower housing demand. One of the central questions of this analysis is the extent to which the percent of foreclosed properties (as proxied by Property Tax Foreclosure) in a neighborhood relate to the likelihood of other homeowners experiencing Property Tax Foreclosure compared to the effect of merely the absolute number of Property Tax Foreclosures. From the original dataset, I calculated the number of homes on each block that had experienced Property Tax Foreclosure each year (excluding each examined property from its own total). I applied this absolute number as a variable to each property. I then additionally calculated the percent of homes that had experienced Property Tax Foreclosure each year and applied this percent as a variable to each property. I created four sets of these two variables for each property-year, one same-year effect, and three lagged effects (e.g., A 1-year lagged effect for the absolute number of Property Tax Foreclosures constitutes the absolute number of Property Tax Foreclosures 1 year earlier). Additionally, I interacted the neighborhood percentage non-Hispanic white with these variables since literature has identified race (and socioeconomic status) as meaningful in how neighborhoods change due to foreclosure, vacancy and blight.

I performed a Proportional Hazards Test on my Cox model and found many of my covariates to violate the Proportional Hazards Assumption. I examined the shape of the variables as they related to time and decided to handle them by interacting them with the year.

#### Mathematical Simulations

Presuming, Property Tax Foreclosure spatially perpetuates itself, utilizing coefficient estimates from the Cox model in application to Interactive Markov Models, I can simulate how Property Tax Foreclosures will manifest across a neighborhood over time. Interactive Markov Models constitute a mathematical method for simulating how events transpire over time, given that the probability of an event occurring being only dependent on the system's current state (Conlisk, 1976). In this particular simulation, the probability an event occurring is partially dependent on the order of recent events as well.

$$\begin{array}{l} P\left(X_{y}\right)=\left(P*F_{Three}\left(n_{y-3}\right)*F_{Two}\left(n_{y-2}\right)*F_{One}\left(n_{y-1}\right)\right) \end{array}$$

Where *P*(*X*_*y*_) represents the probability that property *X* experiences PTF in step *y*, *P* is the baseline Hazard, *F*_*Three*_(*n*_*y*−3_) is the effect of n other properties on the block experiencing PTF in step *y* − 3, *F*_*Two*_(*n*_*y*−2_) is the effect of n other properties on the block experiencing PTF in step *y* − 2, *F*_*One*_(*n*_*y*−1_) is the effect of n other properties on the block experiencing PTF in step *y* − 1.

I apply my Cox Model to the Interactive Markov Models in a simplified way, only utilizing Hazard Ratios from my model that are relevant to the experiment, excluding control variables and time-interactions. I combine three significant predictors of Property Tax Foreclosure based on the Cox Model, the number of properties on the block that experienced Property Tax Foreclosure three, two, and 1 year earlier.

At the beginning of every step, every property in this simulation (that has not already experienced PTF) has a probability of experiencing Property Tax Foreclosure based on the baseline hazard (0.01) as well as the effect of the number (absolute and percentage) of Property Tax Foreclosures on the properties block in the prior three steps (1, 2, and 3-year lag effects) and the percentage non-Hispanic White and their interactions (using the Hazard Ratios from **Table 5**). Properties within the simulation then experience Property Tax Foreclosure based on these probabilities. Once a property experiences Property Tax Foreclosure, it is no longer capable of experiencing Property Tax Foreclosure again.

I utilize probabilities based on the Cox model (**Table 5**). To see how Property Tax Foreclosures manifest across a number of scenarios, I allow some parameters to vary. Constant, however, across all scenarios, my simulated neighborhood consists of 100 blocks, each made up of 20 properties. These numbers fit with roughly the average size of a census tract and blocks in Detroit. Since I observed neighborhood racial composition to affect the likelihood of Property Tax Foreclosure, I run a scenario where the neighborhood is 0% white and 50% white. While no census tract is 0% white in Detroit, many are very close. I also use a 50% white neighborhood because it captures an opposite extreme of what would likely be considered a fairly-white neighborhood in Detroit. The second parameter I allow to vary is the number of Property Tax Foreclosures I force to have occurred in the three “steps” before the simulation begins. I do this to simulate how differences in the number of Property Tax Foreclosures that occur early on in a neighborhood can affect the neighborhood's long-term trajectory. To represent a scenario where the neighborhood experiences a lot of Property Tax Foreclosure initially, I have it start with 60 Property Tax Foreclosures, 20 in each of the first 3 years. In the opposing scenario where the neighborhood experiences few Property Tax Foreclosures initially, I have it start with 30 Property Tax Foreclosures, 10 in each of the first 3 years. The final parameter I allow to vary is how those initial Property Tax Foreclosures are distributed across a neighborhood. The Cox model indicates a concentration of Property Tax Foreclosures on a block may have a relatively smaller effect than if an equal number of Property Tax Foreclosures were present on a wider number of blocks. To simulate a neighborhood where Property Tax Foreclosures are initially concentrated, every block that I force to experience a Property Tax Foreclosures experiences two Property Tax Foreclosures (though the simulation has the same total number). To simulate a neighborhood where Property Tax Foreclosures are initially dispersed, on every block that I force to experience a Property Tax Foreclosures only experiences one Property Tax Foreclosures. The intersection of these three parameters creates eight distinct scenarios. I simulate over these scenarios 100 times each.

#### Examining Racial Demographic Change

Given my hypothesis regarding how Property Tax Foreclosures have differential impacts based on the racial demographics of a neighborhood, it may be useful to analyze whether initial Property Tax Foreclosures predict racial demographic change in a neighborhood. Notably, the racial composition, in conjunction with the number of initial Property Tax Foreclosures, may be key in determining the number of Property Tax Foreclosures in the long-term. Based on earlier literature, the total number of Property Tax Foreclosed homes is racially meaningful as whites are disproportionality less willing to live in racially mixed neighborhoods that with higher numbers of vacant or dilapidated homes and have greater financial means by which to achieve residence in other neighborhoods. This suggests that whites may be disproportionality more likely to leave a racially-diverse neighborhood if it experiences a greater number of Property Tax Foreclosures, which is partially determined by the initial number of Property Tax Foreclosures and how they are distributed. Racially-diverse neighborhoods are particularly significant in Detroit due to the high total black population percentage in the city resulting in there not existing a census tract in the city that is over 80% non-Hispanic White and the vast majority of non-Hispanic white residents living in the city residing in neighborhoods with fairly high percentages of black residents. Non-Hispanic White residents have left the city at a disproportionately high rate over the last few decades, and so the pattern of how and why they do leave the city is particularly meaningful to the persistence and growth of racial residential segregation in Detroit.

To analyze how Property Tax Foreclosures related to neighborhood racial demographic change, I combined data from the 2000 Census, and the 2013–2017 American Community Survey 5-year estimates. I choose this time range of data because it is the smallest time range in which the entire foreclosure data I examined (2002–2013) was included. I operationalize a “neighborhood” (the geographical unit of analysis) as a census tract. The number of observations I include in my OLS model is subsequently 293 (referring to the 293 census tracts in the city of Detroit).

## Results

### Cox Model

I produced three Cox Models relating to the data. The first Cox Model (Table 3) includes a simultaneous and 3-year lag effect. I find a simultaneous (no-lag) effect of the percentage of properties on the block in interaction with the neighborhood percentage white increases a property's likelihood of experiencing Property Tax Foreclosure. In simpler terms, a property with a higher percentage of neighbors experiencing Property Tax Foreclosure is more likely to experience Property Tax Foreclosure. Properties in whiter neighborhoods are less likely to experience Property Tax Foreclosure, but as more of their block-neighbors experience Property Tax Foreclosure, this disparity diminishes. Since Property Tax Foreclosure is preceded by 3 years of failure to pay Property Taxes, this finding indicates that when a property owner's neighbors stop paying Property Taxes and possibly even vacate their property (presumably 3 years earlier), there is an immediate increase in the likelihood that nearby property owners are not paying Property Taxes or may even be vacating their property, resulting in the observed effect 3 years later. (This finding follows sensibly from the basic expectation of positive spatial autocorrelation). The interaction of this variable with neighborhood percentage white follows what I would expect based on past literature since, as discussed earlier, White property owners have greater residential preference differences between non-blighted racially diverse neighborhoods and blighted-racially diverse neighborhoods, as well as that vacated properties, have stronger localized effects in higher-socioeconomic status neighborhoods (whiter neighborhoods tend to have higher-socioeconomic status). The finding that this variable is positively significant as a percentage, rather than an absolute number, potentially speaks to the non-discrete effect of foreclosed homes being vacant/blighted properties. Vacant and blighted properties only affect nearby properties to the extent by which they appear vacant/unkempt (Whitaker and Fitzpatrick, 2013), so a percentage sensibly best determines how vacant homes may relate to the lack of desirability of continuing to reside in the neighborhood.

**Table 3:** Cox model one.

**Variable**	**Hazard ratio**	**Z**
Percent Block Properties Exp. PTF	25.45594^***^	(8.30)
Percent non-Hispanic white	0.9886637^***^	(−12.14)
Percent Block Properties Exp. PTF X percent non-Hispanic white	1.026715^***^	(5.52)
Abs. # of Block Prop. Exp. PTF	1.662645^***^	(19.32)
Abs. # of Block Prop. Exp. PTF X percent non-Hispanic white	0.999059^***^	(−3.67)
Abs. # of Block Prop. Exp. PTF - 3 year Lag	1.393427	(1.69)
Abs. # of Block Prop. Exp. PTF - 3 year Lag X % non-Hispanic white	0.9999402	(−0.09)
Percent of Block Prop. Exp. PTF - 3-year Lag	3.509464	(0.49)
Percent of Block Prop. Exp. PTF - 3-year Lag X percent non-Hispanic white	1.020575	(1.65)
Observations	2,756,508	

^*^p < 0.05, ^**^p < 0.01,

*** *p < 0.001*.

*Other variables included in the model but for which Hazard Ratios are not listed for include Total Number of Properties on Block, Proportion of Vacant Properties for Rent, Proportion of Vacant Properties for Sale, Proportion of Occupied Units Owner-Occupied, Proportion of Residents in Poverty, Proportion of Housing Unites with Mortgage > 30% of Income, Renter Occupied Housing Costs, Percent of Residents that are Employed, Percent of Residents in Labor Force. All (non-interaction) variables were interacted with year so as to maintain proportional hazards*.

Tables 4, 5 depict the second and third models, respectively. These models exclude the simultaneous effect in order to more clearly examine the lagged effects (since the simultaneous effect to some extent would be expected to mediate the lagged covariates). Another significant finding is the absolute number of Property Tax Foreclosures on a block 3 years earlier increases the likelihood that a property will experience Property Tax Foreclosure. Three years is the number of years it takes for Property Tax Foreclosure to occur once a property owner vacates a property/stops paying Property Tax. This suggests for this effect, the impetus for vacating a property/stopping paying Property Tax is the act of Property Tax Foreclosure (since the vacating of other properties/failure to pay property tax would have occurred 6 years earlier). As Whitaker and Fitzpatrick (2013) state, the actual action of Property Tax Foreclosure can have distinct effects on the surrounding area, as it could potentially be a highly-visible event and also potentially lead to reappraisal and revaluation of nearby homes, both of which affects residents desire to continue to reside at their current property. This effect sensibly interacts with the Neighborhood percentage non-Hispanic White for the same reasons discussed in the last effect. The finding that this effect is a result of the absolute number of properties that experienced Property Tax Foreclosure 3 years earlier rather than a percentage speaks to the possibly more discrete effect of the action of Property Tax Foreclosure, where even one Property Tax Foreclosure on a block with a high number of total properties can have a substantial effect.

**Table 4:** Cox model two.

**Variable**	**Hazard ratio**	**Z**
Percent non-Hispanic white	0.989037^***^	(−12.54)
Abs. # of Block Prop. Exp. PTF - 3 year Lag	1.903586^**^	(3.04)
Abs. # of Block Prop. Exp. PTF - 3 year Lag X % non-Hispanic white	1.001235	(1.79)
Percent of Block Prop. Exp. PTF - 3-year Lag	0.9048883	(−0.03)
Percent of Block Prop. Exp. PTF - 3-year Lag X percent non-Hispanic white	1.002933	(0.23)
Observations	2,756,508	

^*^p < 0.05,

** *p < 0.01*,

*** *p < 0.001*.

*Other variables included in the model but for which Hazard Ratios are not listed for include Total Number of Properties on Block, Proportion of Vacant Properties for Rent, Proportion of Vacant Properties for Sale, Proportion of Occupied Units Owner-Occupied, Proportion of Residents in Poverty, Proportion of Housing Unites with Mortgage > 30% of Income, Renter Occupied Housing Costs, Percent of Residents that are Employed, Percent of Residents in Labor Force. All variables were interacted with year so as to maintain proportional hazards*.

**Table 5:** Cox model three.

**Variable**	**Hazard ratio**	**Z**
Percent Block Properties Exp. PTF - 1 year Lag	4.673091	(1.70)
Percent non-Hispanic white	0.9881141^***^	(−12.48)
Percent Block Properties Exp. PTF – 1 year Lag X percent non-Hispanic white	1.032545^***^	(4.73)
Abs. # of Block Prop. Exp. PTF – 1 year Lag	1.239516^***^	(3.79)
Abs. # of Block Prop. Exp. PTF – 1 year Lag X percent non-Hispanic white	0.9987436^**^	(−3.39)
Abs. # of Block Prop. Exp. PTF - 2 year Lag	1.569961^***^	(9.12)
Abs. # of Block Prop. Exp. PTF - 2 year Lag X % non-Hispanic white	0.9989999	(−1.87)
Percent of Block Prop. Exp. PTF - 2-year Lag	4.119808	(1.68)
Percent of Block Prop. Exp. PTF - 2-year Lag X Percent non-Hispanic white	1.034956^^***^^	(3.72)
Abs. # of Block Prop. Exp. PTF - 3 year Lag	1.747579^**^	(2.74)
Abs. # of Block Prop. Exp. PTF - 3 year Lag X % non-Hispanic white	1.000677	(0.93)
Percent of Block Prop. Exp. PTF - 3-year Lag	1.511811	(0.15)
Percent of Block Prop. Exp. PTF - 3-year Lag X percent non-Hispanic white	1.005212	(0.41)
Observations	2,756,508	

*p < 0.05,

** *p < 0.01*,

*** *p < 0.001*.

*Other variables included in the model but for which Hazard Ratios are not listed for include Total Number of Properties on Block, Proportion of Vacant Properties for Rent, Proportion of Vacant Properties for Sale, Proportion of Occupied Units Owner-Occupied, Proportion of Residents in Poverty, Proportion of Housing Unites with Mortgage > 30% of Income, Renter Occupied Housing Costs, Percent of Residents that are Employed, Percent of Residents in Labor Force. All variables were interacted with year so as to maintain proportional hazards*.

### Mathematical Simulations

**Figure 2:**
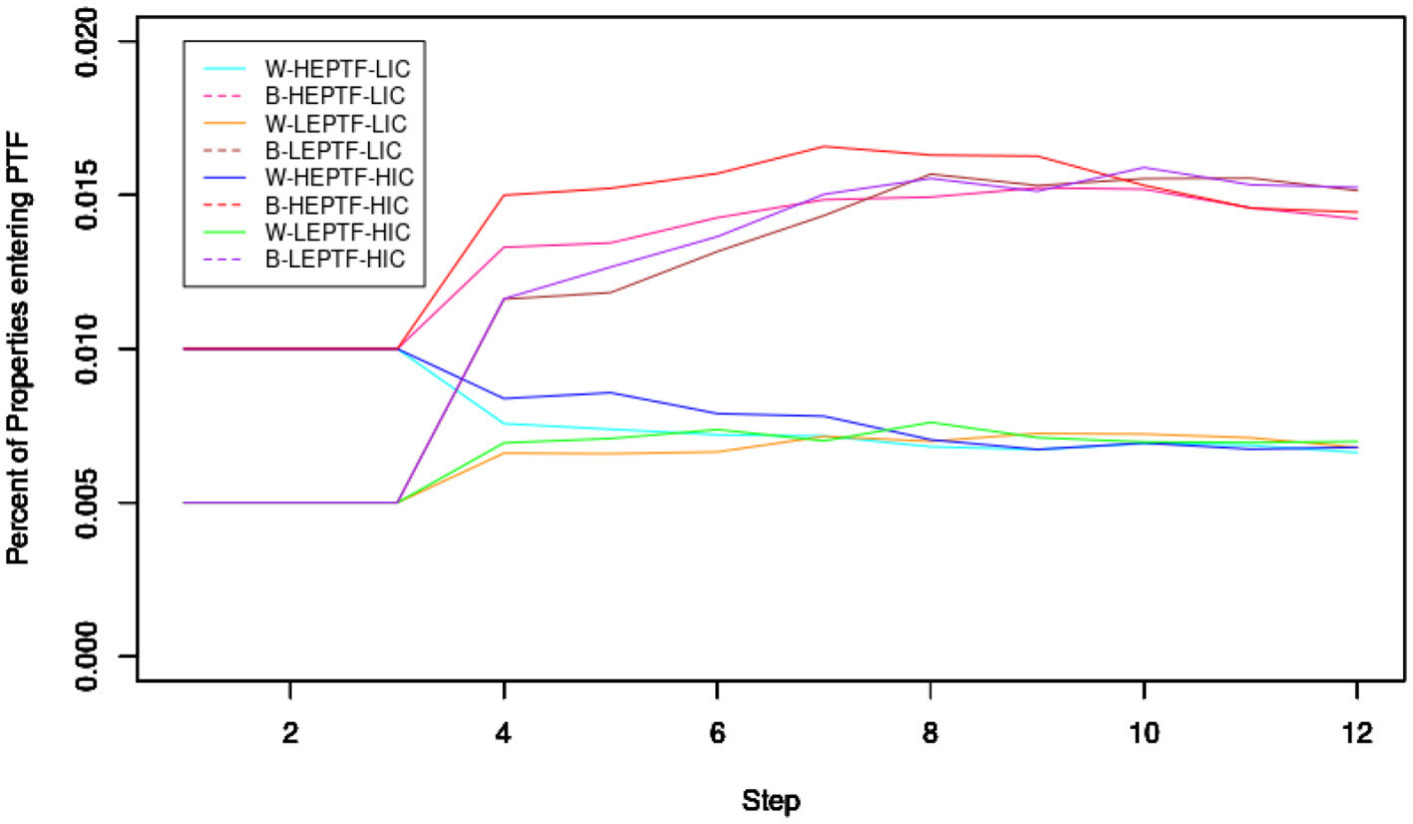
Neighborhood simulation. The Y-axis indicates the total proportion of properties that have experienced Property Tax Foreclosure in that step and the X-axis indicates the step number (e.g., 0.2 on the y-axis means 20%). W indicates that scenario is for a neighborhood that is 50% White, while B indicates the scenario is for a neighborhood that is 0% White. LEPTF indicates that the scenario is for a neighborhood that has a lower number (30) of initial Property Tax Foreclosures. HEPTF indicates that the scenario is for a neighborhood that has a higher number (60) of initial Property Tax Foreclosures. H/L IC indicates that a simulation had a high or low initial concentration of Property Tax Foreclosures.

Figure 2 displays how the total number of Property Tax Foreclosure manifests across the scenarios (described in Methods) over 13 steps (3 pre-determined, 10 determined by the simulation.) As is evident, certain conditions lead to more significant numbers of overall Property Tax Foreclosure. Steps 4–6, the first 3 steps after the pre-determined steps, we see far more Property Tax Foreclosures in the steps with the greater number of initial foreclosures. This disparity gradually disappears as the Steps go on and race becomes the dominant factor. As is expected, racial disparities in Property Tax Foreclosures were smallest in Steps 4–6, indicating that greater numbers of Property Tax Foreclosures in recent years partially mitigate the role of race in predicting Property Tax Foreclosure. Generally, the spatial perpetuation of Property Tax Foreclosures hurts non-white neighborhoods substantially, while differently seeded scenarios of the same racial composition eventually converged, seeding of a greater number of initial Property Tax Foreclosures substantially accelerated foreclosure in the non-white neighborhood simulation. Of additional interest, scenarios where the Property Tax Foreclosures are dispersed only ended up with a slightly different number of total Property Tax Foreclosures, despite the Cox Model suggesting dispersion may matter.

These results, which provide clarity to the Cox model, suggest notions for how we can have expected neighborhoods to have changed in Detroit between 2002 and 2013. First, we can expect neighborhoods that experienced more Property Tax Foreclosures early on to experience more Property Tax Foreclosures overall. Second, we can expect this effect to be more substantial in whiter neighborhoods. Third, based on my initial literature review, given whites' lower preference for neighborhoods with higher numbers of vacant homes coupled with possessing greater social and financial resources with which to be able to access wider-variety of neighborhoods, we can expect whiter neighborhoods that experienced more Property Tax Foreclosures earlier on and subsequently in total to experience the highest level of Racial Demographic Change.

### Neighborhood Racial Demographic Change

My model includes some variables in predicting how Detroit Neighborhoods changed between 2000 and 2017. While I attempted to predict how neighborhood racial composition changed based on how common Property Tax Foreclosures were within the neighborhood within the 2002–2013 period for which data was available, I further attempted to examine effects on how distributed Property Tax Foreclosures were within the neighborhood. My analysis results showed how Property Tax Foreclosures are perpetuated on a “block” scale. Thus, it is fair to hypothesize that neighborhoods with Property Tax Foreclosures distributed across more “blocks” would have more significant effects across the neighborhood.

As Table 6 shows, there is some evidence of the indirect effect of the initial distribution of Property Tax Foreclosure in a neighborhood (% of Blocks w/ ≥ 1 PTF 02-04), in interaction with the initial frequency of Property Tax Foreclosure (PTF Freq. 02-04), on the neighborhood's long-term racial demographic change. In other words, census tracts with the same number of Property Tax Foreclosures, but dispersed to a greater number of blocks experienced greater declines in the proportion of the population that is non-Hispanic white. The additional significance of the initial distribution of Property Tax Foreclosures directly on the long-term number of Property Tax Foreclosures interestingly stands in distinction to what the mathematical simulations indicated, which failed to present any substantial effect. Future research should further investigate the long-term effects of the initial distributions of Property Tax Foreclosures.

**Table 6:** Demographic change model.

	**Model 1**	**Model 2**
	**Neigh. Black % change 00-17**	**Neigh. Black % change 00-17**
2000 black percentage	0.246^***^	0.217^***^
	(4.83)	(4.20)
2000 white percentage	0.723^***^	0.682^***^
	(8.09)	(7.48)
	(2.20)	(4.96)
% of blocks w/ ≥ 1 PTF 02-04	151.0^*^	
	(2.04)	
PTF freq. 02-04	−690.3^***^	
	(−4.08)	
% of blocks w/ ≥ 1 PTF 02-04 X PTF Freq. 02-04	9136.8^**^	
	(2.88)	
Constant	−29.93^***^	−32.04^***^
	(−5.85)	(−6.27)
Observations	293	

* *p < 0.05*,

** *p < 0.01*,

*** *p < 0.001*.

*Model 1 VIF = 1.4301866, Model 2 VIF = 1.3205246*.

*In maintaining consistency with an earlier draft of this paper, I operationalized blocks slightly differently in this model than I did in the Cox Model, separating odd and even addresses into different “blocks.” The interpretation is essentially the same*.

## Conclusion

This paper consists of several empirical analyses examining the phenomena of Property Tax Foreclosure, including how it manifests/perpetuates itself and, subsequently, how it relates to Detroit's demographic change. My Cox Proportional Hazards Models identified the nuanced effects of the spatial perpetuation of Property Tax Foreclosures. My mathematical simulations suggest implications for how these nuanced effects may affect how neighborhoods experience Property Tax Foreclosures in the long-term. Utilizing past literature on residential patterns, I hypothesized implications for how initial Property Tax Foreclosure patterns likely related to long-term demographic change, which I found some evidence for through an empirical analysis of combined Census-American Community Survey data.

There are multiple key findings of the Event History Analysis of Property Tax Foreclosure. I find neighborhood racial composition plays a significant role in how Property Tax Foreclosure manifests and perpetuates. First, the percentage of residents in a neighborhood that are non-Hispanic white is inversely related to a property's probability of experiencing Property Tax Foreclosure. However, the percentage of residents in a neighborhood that are non-Hispanic white is directly related in interaction with the percent of a property's neighbors that have experienced foreclosure to the property's probability of experiencing Property Tax Foreclosure.

Beyond racial composition effects, I find a simultaneous and a lagged effect of neighbor's Property Tax Foreclosure on a property's Hazard probability of experiencing Property Tax Foreclosure. I suggest the simultaneous effect can be explained by the explicit action of vacating properties or discontinuing paying property tax tending to be highly spatially autocorrelated with nearby property owners' likelihood of vacating their property or discontinuing to pay property tax. The 3-year lagged effect can be explained by the notion that it takes 3 years for a Property Tax Foreclosure to occur following a homeowner no longer paying property taxes. Thus, the 3-year lagged Property Tax Foreclosure effect is an effect of the actual Property Tax Foreclosure process rather than the presumed vacating of nearby properties, which would have occurred 6 years earlier.

I next performed mathematical simulations, examining how different initial Property Tax Foreclosures setups manifested more Property Tax Foreclosures across different scenarios. This analysis suggested that the number of initial Property Tax Foreclosures was directly related to the number of total Property Tax Foreclosures in the long-term and that while initial Property Tax Foreclosures especially accelerate more Property Tax Foreclosures in non-white neighborhoods, initial Property Tax Foreclosures additionally reduce some of the long-term gap in Property Tax Foreclosure between non-white and whiter neighborhoods. The simulations notably only found a slight effect of Property Tax Foreclosures' initial dispersion on the number of Property Tax Foreclosures in the long-term.

Past literature has suggested that neighborhood blight and racial diversity are particularly meaningful as they relate to neighborhood preferences and patterns of non-Hispanic Whites (Krysan et al., 2009). I examined empirical data from Detroit from 2000 to 2017, finding that the initial distribution of Property Tax Foreclosures in Detroit played some role in how neighborhoods experienced long-term demographic change. Neighborhoods with greater initial dispersion (in 2004) of Property Tax Foreclosures ended up experiencing more significant increases in the black neighborhood proportion, even after controlling for initial racial demographics. A standard measure for Black-White racial segregation is the average proportion black in the average black resident's neighborhood. This finding, therefore, suggests that greater initial dispersion of Property Tax Foreclosures related to increases in Black Self-Exposure (segregation) within the particular neighborhoods themselves, and presumably at the larger scale of the metropolitan area as a whole since it is unlikely external moves of black residents from these neighborhoods to less black neighborhoods could fully offset this effect. This being said, it is difficult to more clearly link Property Tax Foreclosure in city of Detroit alone to segregation across the metropolitan area as a whole.

These findings suggest implications for urban policy as well as possibilities for future continued research. The actual action of the city putting a property through Property Tax Foreclosures appears to partially responsible for further perpetuating Property Tax Foreclosures. Given a large number of vacant properties in many large American cities, local governments need to re-evaluate whether the action of Property Tax Foreclosure is worth it relative to the high and numerous external costs such large scales of vacant properties can cause. Notably, partially through their Land Bank, the city of Detroit has attempted to reduce the number of Property Tax Foreclosures in recent years, with the number being down to only 4,709 in 2018 and 514 in 2019. This research suggests that fewer Property Tax Foreclosures may have numerous positive impacts on the city, including disproportionality slowing the rate of Property Tax Foreclosure and housing vacancy and slowing the rate of neighborhood racial demographic change. Future policy, not just in Detroit, but across the United States, should consider policy alternatives to Property Tax Foreclosures, including offering would-be foreclosed homeowners the option to agree to monthly payment plans to avoid Property Tax Foreclosure, as Detroit is currently doing. Future research should continue to examine the spatial effects of Property Tax Foreclosure, particularly given the depth of data available, there is substantially more work that can be done.

## Data Availability Statement

Publicly available datasets were analyzed in this study. This data can be found at: https://portal.datadrivendetroit.org/.

## Author Contributions

The author confirms being the sole contributor of this work and has approved it for publication.

## Conflict of Interest

The author declares that the research was conducted in the absence of any commercial or financial relationships that could be construed as a potential conflict of interest.
